# Bax/Bak activation in the absence of Bid, Bim, Puma, and p53

**DOI:** 10.1038/cddis.2016.167

**Published:** 2016-06-16

**Authors:** J Zhang, K Huang, K L O'Neill, X Pang, X Luo

**Affiliations:** 1 Eppley Institute for Research in Cancer and Allied Diseases, Fred & Pamela Buffett Cancer Center, University of Nebraska Medical Center, Omaha, NE 68198-7696, USA; 2 Xiangya Hospital, Central South University, Changsha 410008, China

## Abstract

How BH3-only proteins activate Bax/Bak, the two gateway proteins of the mitochondria-dependent apoptotic pathway, remains incompletely understood. Although all pro-apoptotic BH3-only proteins are known to bind/neutralize the anti-apoptotic Bcl-2 proteins, the three most potent ones, Bid (tBid), Bim, and Puma, possess an additional activity of directly activating Bax/Bak *in vitro*. This latter activity has been proposed to be responsible for triggering Bax/Bak activation following apoptotic stimulation. To test this hypothesis, we generated *Bid*^*−/*^*^−^Bim*^*−/*^*^−^Puma*^*−/*^*^−^* (TKO), TKO*/Bax*^*−/*^*^−^/Bak*^*−/*^*^−^* (PentaKO), and PentaKO*/Mcl-1*^*−/−*^ (HexaKO) HCT116 cells through gene editing. Surprisingly, although the TKO cells were resistant to several apoptotic stimuli, robust apoptosis was induced upon the simultaneous inactivation of Bcl-xL and Mcl-1, two anti-apoptotic Bcl-2 proteins known to suppress Bax/Bak activation and activity. Importantly, such apoptotic activity was completely abolished in the PentaKO cells. In addition, ABT-737, a BH3 mimetic that inhibits Bcl-xL/Bcl-w/Bcl-2, induced Bax activation in HexaKO cells reconstituted with endogenous level of GFP-Bax. Further, by generating TKO*/p53*^*−/−*^ (QKO) cells, we demonstrated that p53, a tumor suppressor postulated to directly activate Bax, is not required for Bid/Bim/Puma-independent Bax/Bak activation. Together, these results strongly suggest that the direct activation activities of Bid (tBid), Bim, Puma, and p53 are not essential for activating Bax/Bak once the anti-apoptotic Bcl-2 proteins are neutralized.

Apoptosis has an important role in shaping body structures during development and maintaining tissue homeostasis in multicellular organisms.^1^ In response to numerous extracellular and intracellular apoptotic signals, cells initiate divergent intracellular pathways, which converge on the mitochondria to trigger mitochondrial outer membrane permeabilization (MOMP).^2^ The Bcl-2 family proteins, which include the anti-apoptotic members (Bcl-2, Bcl-xL, Bcl-w, Mcl-1, and A1), the effector proteins Bax and Bak, and the pro-apoptotic BH3-only proteins (Bad, Bid, Bik, Bim, Bmf, Hrk, Noxa, and Puma), are major regulators and effectors of MOMP.^3, 4, 5^ Whereas the anti-apoptotic members suppress MOMP by inhibiting the activation and activities of Bax/Bak, the pro-apoptotic BH3-only proteins, considered the sentinels for the stress signals, promote MOMP by directly or indirectly activating Bax/Bak.^6^ Once activated, both Bax and Bak form homo-oligomers on the outer mitochondrial membrane (OMM), and apparently generate membrane pores, which allow the escape of apoptogenic proteins, that is, cytochrome C and SMAC, from the intermembrane space, leading to the formation of the apoptosome and the subsequent effector caspase activation.^7^

Bax and Bak are normally globular shaped alpha-helical proteins, both containing a hydrophobic helix surrounded by eight amphipathic helices.^8, 9^ While Bak homo-dimers reside on the OMM, Bax is primarily a cytosolic monomeric protein under normal conditions.^8, 10^ Responding to various apoptotic stimuli, different subsets of BH3-only proteins become transcriptionally or posttranslationally activated (i.e., tBid and Bim).^11, 12, 13, 14, 15^ These activated BH3-only proteins then trigger dramatic conformational changes in Bax and Bak, causing Bax to rapidly move to the OMM, where both Bax and Bak coalesce to generate homo-oligomers necessary for pore formation.^2^ Conceivably, the transition of Bax and Bak from harmless proteins to potent killers represents a critical switch in the mitochondria-dependent apoptotic pathway, and the molecular mechanism of this conversion has remained a major focus in apoptosis research over the past two decades.^16, 17^

A large number of biochemical and structural studies have uncovered three interactions among the Bcl-2 family proteins. First, the anti-apoptotic family proteins interact with active Bax/Bak to inhibit their activity, and the removal of this inhibition is considered a requisite step before Bax/Bak activation.^18, 19^ Second, the activated BH3-only proteins sequester the anti-apoptotic Bcl-2 proteins;^20, 21^ third, three BH3-only proteins, tBid, Bim, and Puma, through their BH3 domains, directly bind and activate Bax/Bak.^22, 23, 24, 25, 26^ On the basis of these interactions, two major models of Bax/Bak activation have been proposed. The Indirect Activation model suggests that Bax/Bak are activated through a relief of inhibition, as the BH3-only proteins bind and neutralize all the anti-apoptotic Bcl-2 proteins, which otherwise sequester active Bax/Bak.^18, 27^ However, such a mechanism has difficulty in explaining the mitochondrial translocation of monomeric Bax during apoptosis. On the other hand, the Direct Activation model suggests that Bax/Bak are activated by the direct binding of ‘direct activator' BH3-only proteins, for example, tBid and Bim, and such activation is facilitated by sensitizer BH3-only proteins, for example, Bad, which neutralize anti-apoptotic Bcl-2 proteins, freeing the direct activators.^22, 25, 28^ In addition to the BH3-only proteins, the tumor suppressor p53 has also been found to possess the activity of directly activating Bax.^29, 30^ Recent studies demonstrated that the DNA binding domain of p53 directly binds to the hydrophobic groove of Bax, and this interaction was postulated to directly trigger the conformational changes of Bax needed for its activation.^31^

Despite compelling data from *in vitro* studies, the physiological relevance of such a direct activation mechanism has not been established.^32, 33^ In this study, genetic approaches were used to investigate the role of the three major direct activator BH3-only proteins Bid, Bim, and Puma, and the tumor suppressor p53 in Bax/Bak activation during apoptosis.

## Results

The direct activator BH3-only proteins not only possess the ability to directly activate Bax/Bak, but also avidly bind to and neutralize the anti-apoptotic Bcl-2 proteins.^34^ Thus, a genetic elimination of a direct activator BH3-only protein will result in the simultaneous loss of both activities. To evaluate the roles of the neutralizing and direct activating activities of the direct activators, we used a two-step approach involving the genetic loss of the direct activators and the genetic or functional ablation of the anti-apoptotic Bcl-2 proteins. First, we generated cells deficient for all direct activators, effectively eliminating both activities of these proteins. Second, we restored the neutralizing activity of the direct activators in these knockout cells with a combination of genetic ablation, siRNA knockdown, and/or specific inhibitor of the anti-apoptotic Bcl-2 proteins, essentially mimicking the neutralizing activity of the direct activators in the absence of these proteins. This system allowed us to assess the contributions of the neutralizing and direct activating activities of the direct activator BH3-only proteins in Bax/Bak activation.

### Generation of the TKO and PentaKO cell

**Figure 1:**
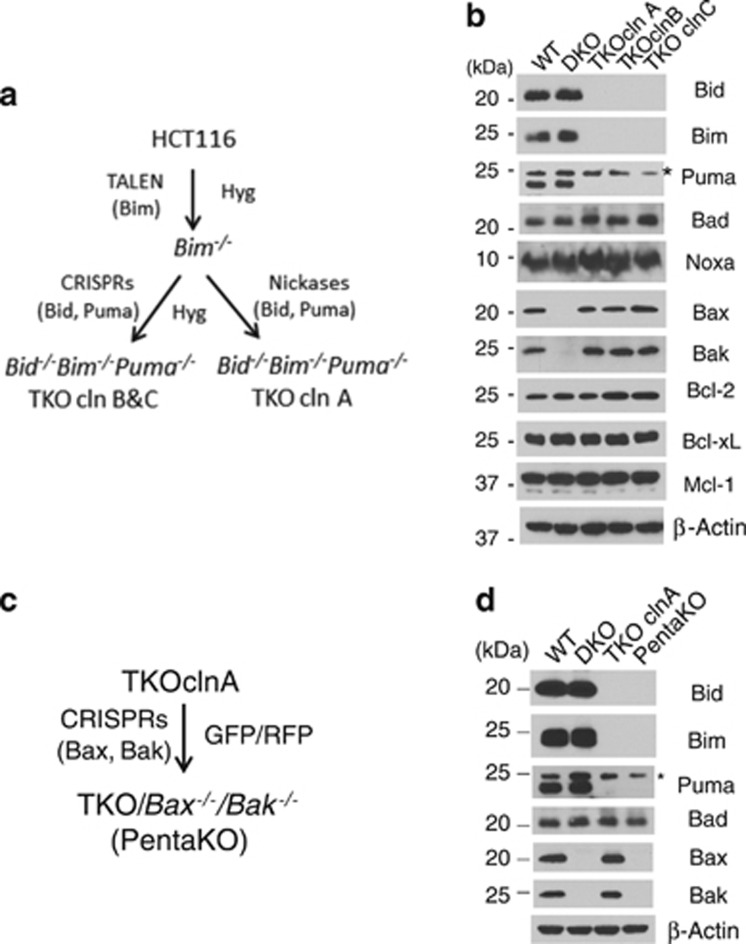
Generation of the TKO and PentaKO HCT116 cells. (**a**) Diagram of the strategy for the generation of *Bid*^*−/−*^ *Bim*^*−/−*^*Puma*^*−/−*^ HCT116 cells. (**b**) Western blot of the cell lysates from the three TKO clones. * indicates a non-specific protein. (**c**) Diagram of the strategy for the generation of TKO/*Bax*^*−/−*^*Bak*^*−/−*^ (PentaKO) HCT116 cells. (**d**) Whole cell lysates of the indicated cell lines were Western blotted against the indicated antibodies

To investigate the role of the three commonly recognized direct activator BH3-only proteins, Bim, Bid (tBid), and Puma, in Bax/Bak activation, a combination of transcription activator-like effector nucleases (TALEN)^35^ and CRISPR-Cas9^36, 37^ techniques was used to generate human colon cancer HCT116 cells deficient for all three proteins. First, a TALEN-expressing plasmid that targets the first exon of Bim was constructed and introduced into HCT116 cells by transient transfection together with a reporter plasmid expressing RFP and GFP-hygromycin resistance fusion protein (G-HygR) as an indicator for the transient expression of the transfected TALEN. Following a selection by hygromycin and subsequent screening by western blot, three Bim-deficient clones were isolated, mixed, and transfected with two CRISPR-expressing plasmids (Strategy 1), or two pairs of Nickase (a variant of CRISPR)-expressing plasmids (Strategy 2), targeting the genomic sequences upstream from the respective BH3 domains of Bid and Puma, separately (Figure 1a). Following another hygromycin selection, single clones from each strategy were screened for the loss of both Bid and Puma proteins. Three clones of *Bid*^*−/−*^*Bim*^*−/−*^*Puma*^*−/−*^ triple knockout cells (named TKOs), two from strategy 1 and one from strategy 2, were confirmed by western blot (Figure 1b) and genomic sequencing (Supplementary Figures S1 and Supplementary Table S1). Similarly, CRISPR-expressing plasmids targeting Bax and Bak, separately, were constructed and introduced into HCT116 cells to generate *Bax*^*−/−*^*Bak*^*−/−*^ double knockout cells (named DKOs, Figure 1b).

We also envisioned that if there is Bid/Bim/Puma-independent apoptosis, it will be important to test whether it is Bax/Bak-dependent. We therefore set out to generate Bid/Bim/Puma/Bax/Bak-deficient cells by gene editing. Cells from the TKO clone A were co-transfected with CRISPR-expressing plasmids targeting Bax and Bak. A clone that lost the expression of all five proteins was verified by both western blot analysis and genomic sequencing, and was named the PentaKO cells (Figures 1c and d,Supplementary Figure S1, and Supplementary Table S1).

### TKO cells are resistant to several common apoptotic stimuli

**Figure 2:**
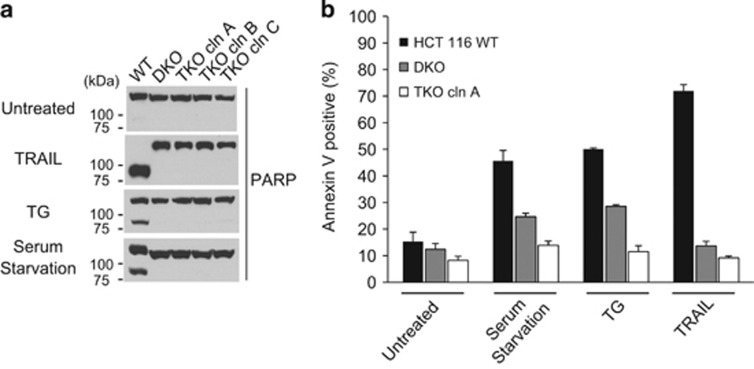
Loss of Bid, Bim, and Puma suppresses apoptosis induced by multiple stimuli. (**a**) The wild-type and the mutant clones of HCT116 were treated with TRAIL (25 ng/ml) for 5 h or with Thapsigargin (3 *μ*M) for 24 h or serum starved for 48 h. Following each treatment, cell lysates were western blotted against an anti-PARP antibody. A representative of three independent experiments is shown. (**b**) The indicated cells were treated by the same apoptotic stimuli as in (**a**), and were stained with Annexin V followed by flow cytometry analysis. Each data point is mean +/− S.E.M. from at least three independent experiments

The three independent clones of the HCT116 TKO cells, along with one of the clones from the DKO cells, were examined for their response to several common apoptotic stimuli, including the death receptor ligand TRAIL, the ER stress inducer Thapsigargin (TG), and serum starvation. Not surprisingly, unlike the wild-type HCT116 cells, both DKO and TKO cells were highly resistant to all three treatments, as evidenced by the lack of PARP cleavage (Figure 2a) and Annexin V signals (Figure 2b) following each treatment. These results demonstrate that the loss of Bid, Bim, and Puma confers resistance to apoptosis induced by TRAIL, Thapsigargin, and serum starvation in HCT116 cells, consistent with findings from an earlier study in which the *Bid*^*−/−*^*Bim*^*−/−*^*Puma*^*−/−*^ mouse embryo fibroblasts were used.^38^

### Suppression of Bcl-xL and Mcl-1 causes Bax/Bak-dependent apoptosis in the TKO cells

**Figure 3:**
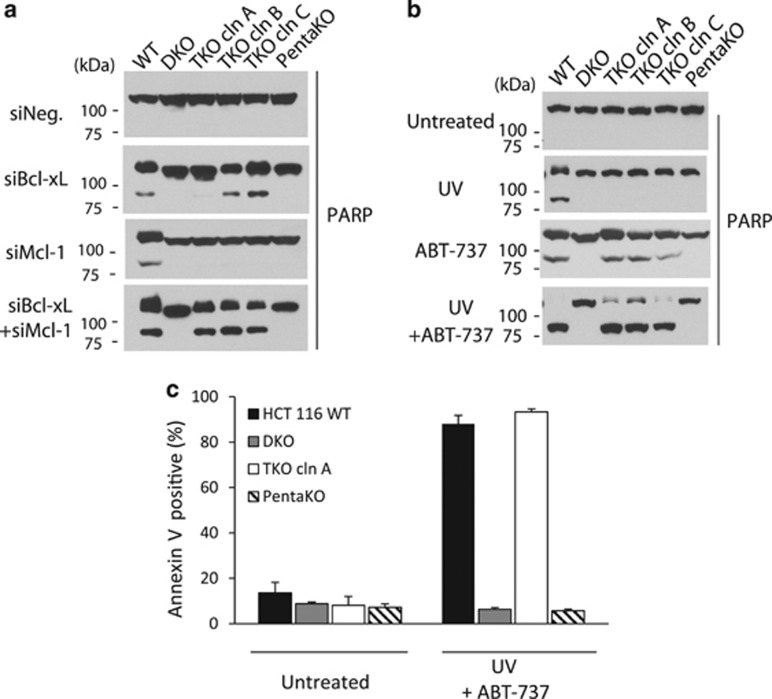
Bax/Bak-dependent apoptosis in the absence of Bid, Bim, and Puma in HCT116 cells following suppression of Bcl-xL and Mcl-1. (**a**) Cell lysates were harvested following transfection by the indicated siRNA oligos as described in Materials and Methods, and western blotted with anti-PARP antibody. (**b**) Cells were treated by either UV (500J/M^2^) or ABT-737 (2.5 *μ*M) or both. Sixteen hours later, cell lysates were harvested and western blotted with anti-PARP antibody. (**c**) Cells were treated by the combination of UV and ABT-737 for 16 h, the same as in (**b**), and were stained with Annexin V followed by flow cytometry analysis. The results are the mean +/− S.E.M. of at least three independent experiments

According to the direct activation model, although all BH3-only proteins can inactivate/neutralize anti-apoptotic Bcl-2 members, only tBid, Bim, and Puma have the additional function of directly engaging and activating Bax/Bak.^25, 28^ It predicts that Bax/Bak activation following neutralization of the anti-apoptotic proteins requires at least one of the three direct activating BH3-only proteins. In an earlier study, inactivation of both Bcl-xL and Mcl-1, mimicked by the simultaneous siRNA knockdown of these two proteins, caused robust apoptosis in HeLa cells.^39^ Using the same approach, the wild-type, DKO, and TKO HCT116 cells were transfected with siRNAs against Bcl-xL and Mcl-1, either singly or in combination (Supplementary Figure S3). Surprisingly, the double knockdown of Bcl-xL and Mcl-1 induced robust apoptosis in both wild-type and TKO cells, but not in DKO or PentaKO cells, indicating that apoptosis in the TKO cells is dependent on Bax/Bak activation (Figure 3a).

As an alternative way to eliminate/inactivate Mcl-1 and Bcl-xL, we also treated the cells with a combination of ultraviolet light (UV) and ABT-737, with ABT-737 added immediately following UV treatment. While UV is known to eliminate Mcl-1 efficiently through a rapid, proteasome-mediated degradation,^40^ ABT-737 is a highly specific BH3 mimetic that potently inhibits Bcl-xL/Bcl-2 /Bcl-w.^41^ As expected, Mcl-1 is essentially eliminated from the wild-type, DKO, TKO, and PentaKO cells 5 h after UV treatment (Supplementary Figure S4). Although the single treatment by ABT-737 caused modest amount of apoptosis, the combination treatment resulted in much stronger apoptosis in both wild-type and TKO cells, as evidenced by the disappearance of the full length PARP protein and the positive Annexin V staining (Figures 3b and c). Of importance, neither single nor combinatorial treatments caused apoptosis in DKO or PentaKO cells. Together, these results indicate that inactivation of both Bcl-xL and Mcl-1 leads to activation of Bax/Bak in the absence of the three direct activator BH3-only proteins, Bid, Bim, and Puma.

### Suppression of Bcl-xL/Bcl-2/Bcl-w and Mcl-1 activates Bax in the absence of Bid, Bim, Puma, and Bak

**Figure 4:**
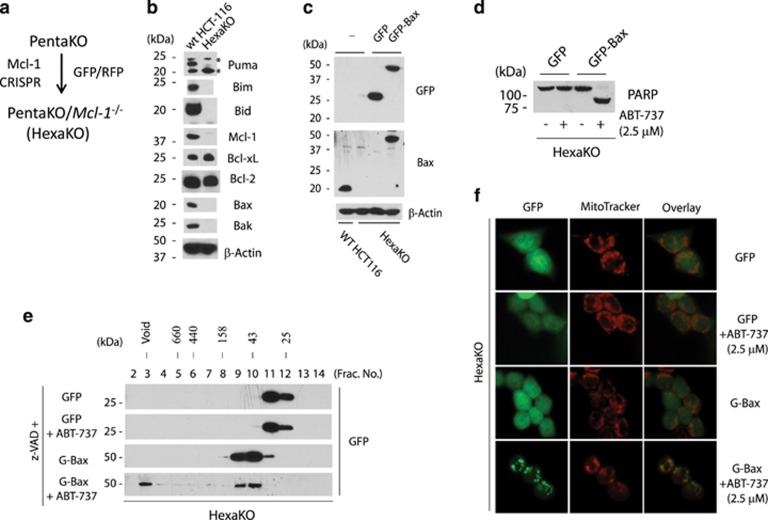
ABT-737 induces Bax activation in the absence of Bid, Bim, Puma, Bak, and Mcl-1. (**a**) Diagram for generating HexaKO cells. (**b**) Western blot of the cell lysates from the wild-type and HexaKO cells. * indicates a non-specific protein. (**c**) Expression level of GFP-Bax in the indicated cells. Cell lysates were western blotted against an anti-Bax or anti-GFP antibody. (**d**) HexaKO cells reconstituted with GFP or GFP-Bax were treated with ABT-737 (2.5 μM) for 6 h. The cell lysates were western blotted with anti-PARP antibody. (**e**) HexaKO cells were treated with ABT-737 (2.5 *μ*M) in the presence of z-VAD (50 *μ*M) for six hours before they were harvested. These cells were lysed in buffer A with 2% CHAPS and loaded onto a Superdex 200 column for gel-filtration analysis. Fractions were western blotted against an anti-GFP antibody. (**f**) HexaKO cells were treated with ABT-737 in the presence of Z-VAD for 6 h and then stained with MitoTracker and photographed under a fluorescence microscope

Under normal conditions, Bax exists in the cytosol as a monomeric and inactive protein. Following apoptotic stimulation, Bax moves to the mitochondria and forms homo-oligomers in the mitochondrial outer membrane.^42^ The mechanism of this translocation remains not well defined. To see whether mitochondrial translocation of Bax can be induced by suppression of the anti-apoptotic members of the Bcl-2 family proteins, we established PentaKO cell pools that either express GFP or those that express GFP-Bax at the endogenous level, and examined the effect of simultaneous suppression of Bcl-xL and Mcl-1. However, owing to the slow kinetics of siRNA transfection and UV treatment, it is difficult to monitor the movement of Bax in these cells. Therefore, we decided to generate Mcl-1-deficient cells by gene editing, and subsequently inhibit Bcl-xL/Bcl-2/Bcl-w by ABT-737. PentaKO cells were transiently transfected with the CRISPR-expressing plasmids against Mcl-1, and the transfected cells were sorted by flow cytometry. A single clone of PentaKO*/Mcl-1*^*−/−*^ (named HexaKO) cells was isolated, and verified by western blot and genomic sequencing (Figures 4a and b,Supplementary Figure S5, and Supplementary Table S1).

HexaKO cells stably expressing either GFP or GFP-Bax were generated by retroviral infection, and subsequently sorted by flow cytometry. The level of GFP-Bax expression in the resulting pool was comparable with that of the endogenous Bax in wild-type HCT116 cells (Figure 4c). The addition of ABT-737 resulted in robust apoptosis and homo-oligomerization as monitored by gel-filtration analysis in the presence of CHAPS^43^ in HexaKO cells expressing GFP-Bax, but not in those expressing GFP, indicating that GFP-Bax was fully functional in these cells (Figures 4d and e). Importantly, this result indicates that apoptosis in HexaKO cells following neutralization of Bcl-xL/Bcl-2/Bcl-w and Mcl-1 is strictly dependent on GFP-Bax. Not surprisingly, although GFP-Bax is predominantly cytoplasmic in heathy HexaKO cells, it efficiently moved to the mitochondria within 6 h following the addition of ABT-737 (Figure 4f), indicating that suppression of the anti-apoptotic Bcl-2 family proteins is sufficient to trigger Bax translocation in the absence of Bid, Bim, Puma, and Bak.

### Neutralization of Bcl-xL and Mcl-1 causes apoptosis in the absence of Bid, Bim, Puma, and p53

**Figure 5:**
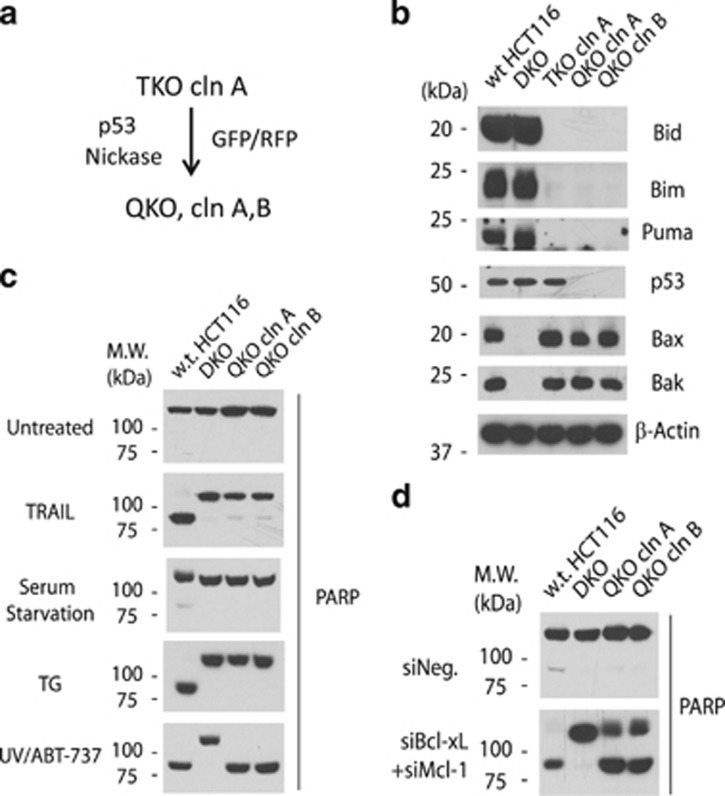
Suppression of Bcl-xL and Mcl-1 causes apoptosis in the absence of Bid, Bim, Puma, and p53. (**a**) Diagram for the generation of Bid/Bim/Puma/p53 QKO cells. (**b**) Western blot of the indicated cell lines. * indicates a non-specific protein. (**c**) The indicated cells were treated with TRAIL (25 ng/ml) for 5 h or with Thapsigargin (3 *μ*M) for 24 h or serum starved for 48 h or treated with combination of UV (500 J/M^2^) and ABT-737 (2.5 *μ*M) for 16 h. Following the indicated treatments, cell lysates were generated for western blot with anti-PARP antibody. (**d**) Cells were harvested following siRNA transfection for western blot with anti-PARP antibody. A representative of three independent experiments is shown

Results from Figures 3 and 4 suggested that the neutralization function of the BH3-only proteins is sufficient for triggering Bax/Bak activation. How are Bax/Bak activated following inactivation of the anti-apoptotic Bcl-2 proteins? As tumor suppressor p53 has been shown to engage and activate Bax/Bak *in vitro*,^30, 44^ it is possible that p53 directly activates Bax and Bak in the TKO cells following Bcl-xL/Mcl-1 inactivation. We therefore sought to eliminate p53 from the TKO cells by constructing a pair of nickase-expressing plasmids^37, 45^ targeting the p53 gene, and co-transfecting into the TKO cells (clone A) (Figure 5a). Following sorting by flow cytometry, two clones of TKO/p53^*−/−*^ (QKO) cells were isolated and validated by western blot and genomic sequencing (Figure 5b,Supplementary Figure S6, and Supplementary Table S1). As expected, the QKO cells were highly resistant to TRAIL, Thapsigargin, and serum starvation (Figure 5c). However, the UV-ABT-737 combinatorial treatment induced robust apoptosis in the QKO cells (Figure 5c). Similar to TKO cells, the QKO cells underwent robust apoptosis following the double siRNA transfection against Bcl-xL and Mcl-1 (Figure 5d). Together, these results indicate that endogenous p53 is not involved in Bax/Bak activation in the TKO cells following inactivation of anti-apoptotic Bcl-2 proteins.

## Discussion

At some time point following apoptotic stimulation, Bax and Bak, the two requisite effectors of MOMP, suddenly turn from inactive monomers into lethal homo-oligomers on the OMM.^42^ What is the immediate trigger for this swift and decisive transition? The answer to this question has been regarded as the ‘holy grail' of apoptosis research.^3^ The three most potent pro-apoptotic BH3-only proteins, tBid, Bim, and Puma, and the tumor suppressor p53 have been considered the triggering proteins owing to their ability to directly bind/activate Bax and Bak *in vitro*.^46^ In this study, we used a combinatorial genetic approach in human colon cancer cells to examine the role of these four proteins in Bax/Bak activation. Surprisingly, we found that upon suppression of both Bcl-xL and Mcl-1, Bax/Bak become highly active in the absence of all four proteins, indicating that their direct activating activities are not necessary for Bax/Bak activation. Consistent with this conclusion, although a strong interaction was detected between Puma and Bcl-xL, no interaction was detected between Puma and Bax in HCT116 cells.^47^ Further, whereas knock-in mutations of Bax in the homo-oligomerization domain^43^ abolished Bax-dependent apoptotic response in HCT116 cells, mutations that disrupt the putative interface between Bax and the direct activator BH3-only proteins (K21E and D33A) failed to suppress apoptosis.^48^ These results appear to be incompatible with the current hypothesis that Bid, Bim, Puma, and p53 directly activate Bax/Bak during apoptosis. Then, how are Bax/Bak activated?

### The linear model of Bax/Bak activation

**Figure 6:**
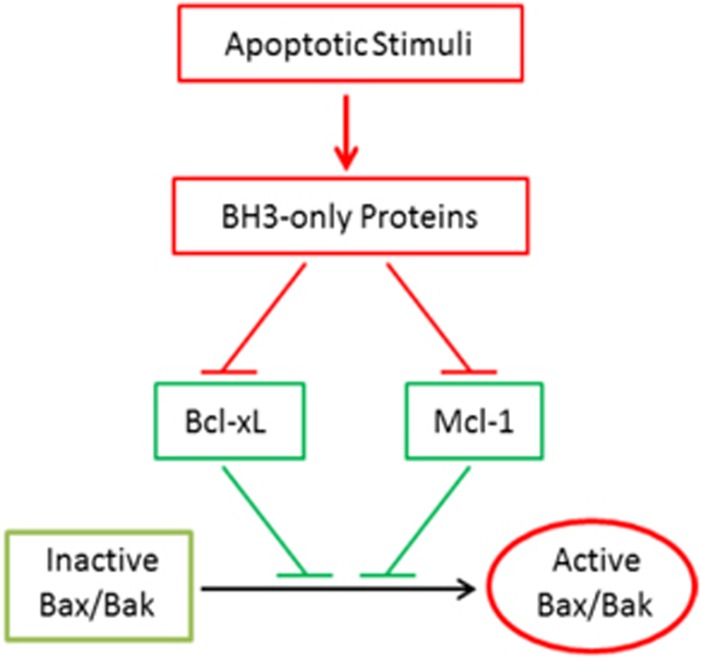
The Linear Model for Bax/Bak activation during apoptosis. In this model, the primary targets of BH3-only proteins are Bcl-xL and Mcl-1, whose primary function is to suppress Bax/Bak activation. Following apoptotic stimulation, the activated BH3-only proteins neutralize/inactivate both Bcl-xL and Mcl-1, allowing Bax and Bak to become activated without the direct activation by BH3-only proteins and p53

Mouse embryonic fibroblasts deficient for Bid, Bim, and Puma have been previously generated and tested for their response to various apoptotic stimuli or overexpression of BH3-only proteins.^27, 38^ However, partly owing to the presence of p53, it has been difficult to assess the role of direct activation *in vivo*. Taking advantage of the TALEN and CRISPR/Cas9 genome editing technology, we established a cellular system in which all three direct activator BH3-only proteins and p53 are eliminated from the same cell. On the basis of the robust apoptosis observed in the QKO cells following the simultaneous suppression of Bcl-xL and Mcl-1 (Figure 5), we propose a Linear Model, in which the major targets of the BH3-only proteins are the anti-apoptotic Bcl-2 proteins, whose primary function is to block the transition of Bax/Bak from inactive to active molecules. During apoptosis, the activated BH3-only proteins neutralize the anti-apoptotic Bcl-2 proteins, and such a functional removal of the Bax/Bak inhibitors then allows Bax/Bak to rapidly transition into active proteins (Figure 6). Although this model is mostly consistent with the traditional Indirect Activation Model in that the primary target of the BH3-only proteins are the anti-apoptotic Bcl-2 family proteins,^21^ it differs from that model in that it does not specify a need for pre-formed complexes between Bax/Bak and the anti-apoptotic Bcl-2 proteins before the onset of apoptosis. Such a distinction accommodates the observation that Bax is normally not on the mitochondria, and through not well understood mechanisms, the mitochondrially localized Bcl-xL retro-translocates Bax to the cytosol.^49^

### How does neutralization of Bcl-xL and Mcl-1 activate Bax/Bak?

As none of the three putative direct activator BH3-only proteins and p53 is required for Bax/Bak activation following inactivation of Bcl-xL and Mcl-1, how then does inactivation of these inhibitors activate Bax/Bak? As active Bak has been found to form complexes with the anti-apoptotic Bcl-2 proteins under normal conditions in some cancer cells, the suppression of Bcl-xL and Mcl-1 is in theory able to liberate the active Bak.^50^ Consistent with the indirect model, Bak was found to be active when anti-apoptotic Bcl-2 proteins were suppressed by siRNA knockdown or small molecule inhibitors in the absence of one, two, or three proteins from the three commonly recognized direct activators Bid, Bim, and Puma.^51, 52^ As for Bax, owing to its cytoplasmic localization, it has been challenging to apply the Indirect Activation Model to Bax's activation. Fletcher *et al.*^18^ proposed that the Bcl-2-like proteins must capture the small proportion of Bax molecules with an exposed BH3 domain, and therefore effectively block the auto-activation mediated through Bax's own BH3 domain.^53^ Alternatively, the inactivation of both Bcl-xL and Mcl-1 resulted in a loss of the fascinating but not well understood retro-translocation activity, which normally shuttles Bax back to the cytoplasm.^49^ A third possibility is that the simultaneous inactivation of Bcl-xL and Mcl-1 causes the release of some positive regulator(s) of Bax/Bak activation.^54, 55, 56^ In addition, recent data found that some other BH3-only proteins, such as Noxa, Hrk, and Bmf, possess weaker direct Bax/Bak activation activities *in vitro*.^57^ Theoretically, it remains possible that one or several of these BH3-only proteins may directly activate Bax/Bak upon release from Bcl-xL/Mcl-1's sequestration in the absence of Bid, Bim, and Puma. However, such a scenario would require a significant revision of the current model for Bax/Bak activation.^46^

### Bax or Bak?

Apoptosis in HCT116 cells following anti-cancer drugs requires only Bax, but not Bak.^58^ This differential requirement has been attributed to the selective suppression of Bak by Mcl-1.^59^ However, it has also been suggested that the direct activator BH3-only proteins selectively activate Bax or Bak in response to chemotherapeutic agents.^60^ The creation of Bid/Bim/Puma TKO, Bid/Bim/Puma/p53 QKO, and PentaKO/Mcl-1^*−/−*^ cells provides useful tools to help resolve the selectivity issue, which is critical in the apoptotic response in cancer following chemotherapeutic treatments.

In summary, our study for the first time demonstrated that inactivation of the two pro-survival Bcl-2 family proteins, Bcl-xL and Mcl-1, triggers Bax/Bak activation in the absence of the three direct activator BH3-only proteins, Bid, Bim, Puma, and the tumor suppressor p53. These data suggest that the direct activation activities of these direct activators are not essential, and that BH3-only protein-mediated neutralization of anti-apoptotic Bcl-2 family proteins serves as the trigger for Bax/Bak activation during apoptosis. These results also highlight the importance of the anti-apoptotic Bcl-2 family proteins as primary targets of therapeutic intervention aimed at potentiating or restoring apoptosis in human cancers, where Bcl-2, Bcl-xL, or Mcl-1 is often overexpressed.

## Materials and Methods

### Cell culture

Cells lines in this study were grown at 37 °C with 5% CO_2_. Cell lines were cultured in media supplemented with 10% fetal bovine serum (Atlanta Biologicals, Flowery Branch, CA, USA, #S11150) and penicillin/streptomycin. HCT116 cells, purchased from ATCC (Manassas, VA, USA), were cultured in McCoy's 5A medium and 293GP cells were cultured in DMEM.

### Reagents

Antibodies used include anti-Bid (Luo *et al.*^12^), anti-Bim (Santa Cruz, Biotechnology, Inc, Dallas, TX, USA, Sc-11425, Calbiochem, San Diego, CA, USA, #202000), anti-Puma (Santa Cruz, Sc-28226, Pro-Sci, Inc, Poway, CA, USA, 3041), anti-Bad (Santa Cruz, Sc-8044), anti-Noxa (Santa Cruz, Sc-56169 Novus Biologicals, Littleton, CO, USA, IMG-349A), anti-Bax (Santa Cruz, Sc-493), anti-Bak (Santa Cruz, Sc-832, Cell Signaling Technology, Danvers, MA, USA, #3814), anti-Bcl-2 (Santa Cruz, Sc-509), anti-Bcl-xL (Santa Cruz, Sc-8392), anti-Mcl-1 (Santa Cruz, Sc-819), anti-*β*-actin (Sigma-Aldrich, St. Louis, MO, USA, A5441), anti-PARP (Cell Signaling Technologies, #9524), anti-GFP (Santa Cruz, Sc-459), and anti-p53 (Santa Cruz, Sc-393). z-VAD(OMe)-FMK was purchased from MP Biomedicals (Santa Ana, CA, USA). Thapsigargin was purchased from Adipogen. Corp (San Diego, CA, USA) ABT-737 was purchased from Cayman Chemical (Ann Arbor, MI, USA). Human recombinant TRAIL was made as previously described.^61^

### Immunoblotting

EBC buffer (50 mM Tris, 120 mM NaCl, 1 mM EDTA, 0.5% NP40, pH=7.5) supplemented with 0.1 mM PMSF and protease inhibitors (5 mg/ml pepstatin A, 10 mg/ml leupeptin) was used to lyse harvested cells with a constant rotation for 1–2 h at 4 °C. Whole cell lysate was collected after centrifugation at 22 000 × *g* for 10 min at 4 °C as the supernatant. Coomassie Protein Assay solution (Thermo Scientific, Waltham, MA, USA, # 1856209) was used to measure protein concentrations of each lysate sample and approximately 50 *μ*g of total protein from each sample was resolved by SDS-PAGE gel. Proteins were then transferred onto a nitrocellulose membrane followed by incubation with primary and secondary antibodies for western blot. Chemiluminescence was used to detect proteins of interest.

### Plasmids

DNA Oligo pairs for sgRNA targeting sites were designed for gene and target regions of interest.^62^ Oligos were annealed and ligated into *Bbs*1 cut px330 or px335 (AddGene, Cambridge, MA, USA, #42230 and #42335). Sequences of oligos that were cloned into px330/px335 are listed in Supplementary Information and Supplementary Table S2. Seoul National University designed and constructed the TALEN expression vector for Bim, TALEN Library Resource (order # H39213).

*Eco*R1 and *Bam*H1 cut sites were used to ligate annealed pairs of oligos containing the TALEN, CRISPR, or Nickase recognition sites into cut reporter plasmid mRFP-TS-2A-HYG-EGFP (PNA Bio Inc, Newbury Park, CA, USA). The following are the sequences ligated into the mRFP-TS-2A-HYG-EGFP: Mcl-1: tctgGTAATAACACCAGTACGGACGGGtcac, Bax: tctcCTGCAGGATGATTGCCGCCGTGGacac, Bak: gggACGGCAGCTCGCCATCATCGGGGacga, Bid: tgcaGCTCATCGTAGCCCTCCCACTGGggag (CRISPR), tcatCCGGAATATTGCCAGGCACCTCGcccaggtcggggacagCATGGACCGTAGCATCCCTCCGGgcct (Nickase), Bim: gatcGCCCAAGAGTTGCGGCGTATTGGagac (CRISPR), TGACCGAGAAGGTAGACAATtgcagc ctgcggAGAGGCCTCCCCAGCTCAGA (TALEN), Puma: ggggCGCTGGGCACGGGCGACTCCA GGtgtc (CRISPR), gggcCCAGCTGCGGCGGATGGCGGACGACCTCAACGCACAGTACGAGCGGcgg (Nickase), p53:tgaaCCATTGTTCAATATCGTCCGGGGacaGCATCAAATCATCCATTGCTTGGgacg (Nickase).

The retroviral expression plasmids pMIG-GFP and pMIG-GFP-Bax were constructed using *Xho*I and *Hind*3 cut sites to ligate DNA fragments containing GFP or GFP-Bax (George *et al.*)^43^ into the *Xho*1/*Hind*3-digested MSCV-IRES-GFP (pMIG) vector. The majority of the sequences encoding the endogenous IRES and GFP in pMIG are abolished by *Xho*1–*Hind*3 digestion.

### Plasmid transfection

Transfection in HCT116 cell transfections were performed with FugeneHD reagent (Promega, Madison, WI, USA, E2311) according to the manufacturer's instructions. CRISPR or Nickase constructs at a concentration of 300–750 ng were co-transfected with 400 ng of the corresponding mRFP-TS-2A-HYG-EGFP reporter plasmid. For TALEN transfection, 1.5 *μ*g of TALEN construct was used along with 400 ng mRFP-TS-2A-HYG-EGFP reporter. A total DNA concentration of 2 *μ*g per transfection was reached by adding pcDNA3.1. Transfected cells were divided into two plates 24 h post transfection. Hygromycin B (1 mg/ml, Calbiochem, #400050) selection or flow cytometry sorting for RFP/GFP-positive cells was performed on transfected cells 30 h post transfection. The selected and sorted cells were subsequently plated for single clones on 15-mm plates.

### siRNA transfection

Cells in 35-mm culture dishes with antibiotic-free McCoy's 5A medium+10% fetal bovine serum and DharmaFECT2 transfection reagent (Dharmacon, Inc., Lafayette, CO, USA, #T-2002-01) were subjected to siRNA transfection. siRNA Buffer (Dharmacon, Inc., #B-002000-UB-100) was used to suspend ON-TARGET Plus siMcl-1, siBcl-xl, or siControl (Dharmacon, Inc., #L-004501-00, #L-003458-00, #D-001810-10-05) at a final concentration of 22 nM. Transfected cells were harvested 48–72 h after transfection for western blot analysis. Sequential transfections were used for double knockdown of Bcl-xl and Mcl-1. The first transfection in cells was with siBcl-xL or siControl. Cells were split into two separate plates 48 h later. For the second transfection, one plate was transfected with siControl RNA and the other with siMcl-1 RNA. Cells subject to double knockdown and the siControl were harvested for western blot analysis with PARP antibody 24 h after the second transfection.

### Flow cytometry

A BD FACSAria or a BD FACSAria II cell sorter with BD FACSDiva 6.1.2 software (BD Biosciences, San Jose, CA, USA) (UNMC, Flow Cytometry Research Facility) was utilized to sort transfected cells according to GFP/RFP profiles. Cells with RFP and/or GFP expression were collected for further analysis and experiments.

### Viral production

pMIG retroviruses were produced in 293GP packaging cells as previously described by Lopez *et al.*^39^ Polybrene (10 ug/ml, American Bioanalytical, Natick, MA, USA, AB01643) was used to carry out viral infection in HCT116 cells.

### Genotyping

DNeasy Blood and Tissue kit (Qiagen, Valencia, CA, USA, #69504) was used to isolate genomic DNA from HCT116 cell lines. Genomic target regions of interest were then amplified via polymerase chain reaction using Phusion or Taq polymerase (New England Biolabs, Ipswich, MA, USA, #E0553S and #M0273L). Genomic PCR primer sequences that were used are listed in Supplementary Information and Supplementary Table S3. T4 ligase was used to clone PCR products into pGEM T-easy vector (Promega, #A1360). Ligated samples were transformed into competent cells and blue/white selection (Thermo Fisher Scientific, Waltham, MA, USA, BP4200-10) was used to pick colonies containing PCR inserts. Miniprep DNA (Wizard Plus SV Minipreps DNA Purification System, Promega, #A1460, Promega) from individual colonies was then digested with *Eco*R1 to determine the size of inserts. Sanger sequencing using T7 or SP6 primers was utilized to obtain insert sequences from purified plasmids (UNMC High-Throughput DNA Sequencing and Genotyping Core Facility). In addition, if the genomic PCR reaction displayed a single band when ran on an agarose gel, direct sequencing of the product using the primers from the original PCR or inner primers was performed to confirm plasmid sequencing results.

### Apoptosis assays

Cells were plated into 35-mm plates 16–20 h prior to treatments. The following reagents were added to plates at the indicated concentrations and durations: Thapsigargin (3 *μ*M) for 24 h, human recombinant TRAIL (25 ng/ml) for 5 h, ABT-737 (2.5 *μ*M) for 16 h, UV (500 J/M^2^) for 16 h or UV/ABT-737 (500J/M^2^-2.5 *μ*M), with ABT-737 (2.5 *μ*M) added immediately following UV treatment for 16 h. Cells were also subjected to serum starvation by placing them in McCoy's 5A media without fetal bovine serum for 48 h. Cells were analyzed by either FACS or western blot against PARP following each treatment. For Annexin V analysis, treated cells were gathered from media in plates and trypsination of attached cells. Annexin V FITC (Biolegend, San Diego, CA, USA, #640906) was used to stain treated cells according to the manufacturer's instructions. Each sample was then analyzed for FITC signal by flow cytometry. A BD FACSCalibur flow cytometer with BD FACSDiva 8.0 software (UNMC, Flow Cytometry Research Facility) was used for cell counting and analysis. Detection of PARP cleavage in treated samples was achieved by preparing whole cell lysates as described above and subjecting lysate to western blot with PARP antibody.

### Mitochondrial staining

HCT116 cells cultured on cover slips were stained with MitoTracker Red CMXRos (Thermo Fisher Scientific, M7512) at a final concentration of 100 nM, followed by a 10–15-min incubation at 37 °C. Following staining, a fluorescence microscope (Nikon Eclips 50i, Tokyo, Japan) was used to take photographs of live cells mounted on glass slides.

### Statistics

For statistical analysis, ANOVA: single factor test was used to assess the significance of mean differences. Differences were considered significant at a *P*-value of 0.05 or less.

## Abbreviations

MOMP: mitochondrial outer membrane permeabilization
Bcl-2: B cell lymphoma 2
OMM: outer mitochondrial membrane
tBid: truncated Bid
TALEN: transcription activator-like effector nuclease
CRISPR: clustered regularly interspaced short palindromic repeats
RFP: red fluorescent protein
GFP: green fluorescent protein
TKO: triple knockout
DKO: double knockout
PentaKO: penta knockout
HexaKO: hexa knockout
QKO: quadruple knockout
TRAIL: TNF-related apoptosis-inducing ligand
ER: endoplasmic reticulum
TG: Thapsigargin
PARP: Poly ADP ribose polymerase
UV: ultraviolet light
FITC: fluorescein isothiocyanate

## Supplementary Material

Supplementary Informations
