# Supplementary material for: Bax/Bak activation in the absence of Bid, Bim, Puma, and p53
Source: Cell Death Dis. 2016 Jun 16;7(6):e2266–. doi: 10.1038/cddis.2016.167 (PMC5143395; doi:10.1038/cddis.2016.167)
Supplement: Supplementary Informations [file cddis2016167x1.pdf]

Fig. S1. Mutations of the targeted Bim, Puma, and Bid loci in the TKO clones and the targeted Bax and Bak loci in DKO and TKO/*Bax*$^{-/-}$*Bak*$^{-/-}$ clones. **a,** Alignment of genomic sequences around the Bim TALEN target region in HCT116 WT and TKO clones A, B, and C. **b,** Alignment of genomic sequences around the Puma Nickase target region in WT and TKO clone A, and the CRISPR target region in WT and TKO clones B and C. **c,** Alignment of genomic sequences around the Bid Nickase target region in WT and TKO clone A and CRISPR target region in WT and TKO clone B and C. **d,** Alignment of genomic sequences around the Bax CRISPR target region in WT, DKO, and TKO/ *Bax*$^{-/-}$*Bak*$^{-/-}$ clones. **e,** Alignment of genomic sequences around the Bak CRISPR target region in WT, DKO, and TKO/ *Bax*$^{-/-}$*Bak*$^{-/-}$ clones. Underlined sequences are the target sequences of TALEN, CRISPRs, or Nickases.

Fig. S2. Inactivation of Puma by a 3 bp in-frame mutation in OctaKO clone A. **a,** Protein sequences of the BH3-domain of the wild-type Puma and mutant Puma$^{\Delta R142}$. **b,** Detection of a 23 kDa protein band (arrow) in whole cell lysates of TKO cln A by a longer exposure of the western blot with the anti-Puma antibody. **c,** Wild-type and DKO cells were transfected with pEGFP-C3, pEGFP-C3-Puma, and pEGFP-C3-Puma$^{\Delta R142}$. Whole cell lysates were collected twenty hours later, and analyzed by western blot with the indicated antibodies.

Fig. S3. SiRNA knockdown of Mcl-1 and/or Bcl-xL in DKO and TKO/*Bax*$^{-/-}$*Bak*$^{-/-}$ cells. **a,** and **b**, DKO (**a**) and TKO/*Bax*$^{-/-}$*Bak*$^{-/-}$ (**b**) cells were harvested and subjected to western blot analysis following siRNA transfection by the indicated siRNA oligos as described in the Methods section.

Fig. S4. Elimination of Mcl-1 in TKO cells following UV treatment. Cells were treated with UV (500 J/m^2) and harvested 5 hours later. Whole cell lysates were analyzed by western blot with the indicated antibodies.

Fig. S5. Mutations of the targeted Mcl-1 locus in HexaKO clone. Genomic Sequence alignment of the Mcl-1 locus around the CRISPR target region in the wild-type HCT116 and HexaKO clone. The CRISPR target sites are underlined.

Fig. S6. Mutations of the targeted p53 loci in QKO cells. Alignment of the genomic sequences of p53 around the Nickase target sites in wild-type HCT116 and the QKO clones. The Nickase target sites are underlined.

a

```
WT Bim_(TL)       CTTCTGATGTAAGTTCTGAGTGTGACCGAGAAGGTAGACAATTGCAGCCTGCGGAGAGGCCTCCCCAGCTCAGACCTGGGGCCCCTACCTCCCT
TKO_clnA_Bim_a    CTTCTGATGTAAGTTCTGAGTGTGACCGAGAAGGTAGACAATTGCAGCC--CGGAGAGGCCTCCCCAGCTCAGACCTGGGGCCCCTACCTCCCT
TKO_clnA_Bim_b    CTTCTGATGTAAGTTCTGAGTGTGACCGAGAAGGTAGACAATT----CCTGCGGAGAGGCCTCCCCAGCTCAGACCTGGGGCCCCTACCTCCCT
TKO_clnB_Bim      CTTCTGATGTAAGTTCTGAGTGTGACCGAGAAGGTAGACAATTG----CTGCGGAGAGGCCTCCCCAGCTCAGACCTGGGGCCCCTACCTCCCT
TKO_clnC_Bim      CTTCTGATGTAAGTTCTGAGTGTGACCGAGAAGGTAGACAATTG----CTGCGGAGAGGCCTCCCCAGCTCAGACCTGGGGCCCCTACCTCCCT
```

b

```
WT Puma_(CR)        CCTGGGTGGGACCGCCCGCCAGAGCCCCGGGGCGCTGGGCACGGGCGACT--CCAGGTGCTGCTCCGCCAGCGAGAGCGAGGG
TKO_clnB_Puma a     CCTGGGTGGGACCGCCCGCCAGAGCCCCGGGGCGCTGGGCACGGGCGACTTTCCAGGTGCTGCTCCGCCAGCGAGAGCGAGGG
TKO_clnB_Puma b     CCTGGGTGGGACCGCCCGCCAGAGCCCCGGGGCG--------------CT--CCAGGTGCTGCTCCGCCAGCGAGAGCGAGGG
TKO_clnC_Puma       CCTGGGTGGGACCGCCCGCCAGAGCCCCGGGGCGCTGGGCACGGGCGA-T--CCAGGTGCTGCTCCGCCAGCGAGAGCGAGGG

WT Puma_(Nickase)   GGGGAGGAGGAACAGTGGCCCGGGAGATCGGGGCCCAGCTGCGGCGGATGGCGGACGACCTCAACGCACAGTACGAGCGGCGG
TKO_clnA_Puma a     GGGGAGGAGGAACAGTGGCCCGGGAGATCGGGGCCCAGCT---GCGGATGGCGGACGACCTCAACGCACAGTACGAGCGGCGG
TKO_clnA_Puma b     GGGGAGGAGGAACAGTGGCCCGGGAGATCGGGGCCCA------------------------CAACGCACAGTACGAGCGGCGG
```

c

```
WT Bid_(CR)         GCTGCTGCGGTTGCCATCAGTCTGCAGCTCATCGTAGCCCTCC-C-ACTGGGGAGCCAGCACTGGCAGCTCGTGGCC
TKO_clnB_Bid a      GCTGCTGCGGTTGCCATCAGTCTGCAGCTCATCGTAGCCCTCC-CTACTGGGGAGCCAGCACTGGCAGCTCGTGGCC
TKO_clnB_Bid b      GCTGCTGCGGTTGCCATCAGTCTGCAGCTCATCGTAGCCCT-------------GCCAGCACTGGCAGCTCGTGGCC
TKO_clnC_Bid        GCTGCTGCGGTTGCCATCAGTCTGCAGCTCATCGTAGCCCTCCAC-ACTGGGGAGCCAGCACTGGCAGCTCGTGGCC

WT Bid (Nickase)    CTGAAAGTCAAGAAGACATCATCCGG---------------------------------------AATATTGCCAGGCACCTCGCCCAGGTCGG
TKO_clnA_Bid a      CTGAAAGTCAAGAAGACATCATCCGG---------------------------------------AATATTGCCAGGCACCTCGCCCAGGTCGG
TKO_clnA_Bid b      CTGAAAGTCAAGAAGACATCATCCGGGGACAGCATGGACCGTAGCATCCTGCATGGACCGTAGCA-------------------------TCGG

WT Bid_(Nickase)    GGACAGCATGGACCGTAGCATCC----------------------------------CTCCGGGCCTGGTGAACGGCCTGGCCCTGCAGCTC
TKO_clnA_Bid a      GG--------------------------------------------------------------------------------CCTGCAGCTC
TKO_clnA_Bid b      GGACAGCATGGACCGTAGCATCCTGCATGGACCGTAGCATCCTGGACCGTAGCATCCCTCCGGGCCTGGTGAACGGCCTGGCCCTGCAGCTC
```

Fig. S1. Mutations of the targeted Bim, Puma, and Bid loci in the TKO clones and the Bax and Bak loci in DKO and TKO/*Bax*$^{-/-}$*Bak*$^{/-}$ (PentaKO) clones.

Continue-

d

WT Bax (CR) TTAGTGTGCGGTGGATGCGGGAATTTTCCACCATCAGCCTGATGCCTGCTCCCCGGCACTGGTTCTCCTCTCTCCTGCAGGATGATTGCCGC--CGTGGACACAGACT
DKO_Bax TTAGTGTGCGGTGGATGCGGGAATTTTCCACCATCAGCCTGATGCCTGCTCCCCGGCACTGGTTCTCCTCTCTCCTGCAGGATGATTGCCGCGCCGTGGACACAGACT
TKO/*Bax*$^{-/-}$*Bak*$^{-/-}$_Bax TTAGTGTG--CACAGACT

e

WT Bak (CR) GGGGCAGGTGGGACGGCAGCTCGCCATC-ATCGGGGACGACATCAACCGACGCTATGACTCAG
DKO_Bak GGGGCAGGTGGGACGGCAGCTCGCCATCAATCGGGGACGACATCAACCGACGCTATGACTCAG
TKO/*Bax*$^{-/-}$*Bak*$^{-/-}$_Bak GGGGCAGGTGGGACGGCAGCTCGCCATCAATCGGGGACGACATCAACCGACGCTATGACTCAG

Fig. S1. Mutations of the targeted Bim, Puma, and Bid loci in the TKO clones and the targeted Bax and Bak loci in DKO and TKO/*Bax*$^{-/-}$*Bak*$^{/-}$ (PentaKO)clones

-continued

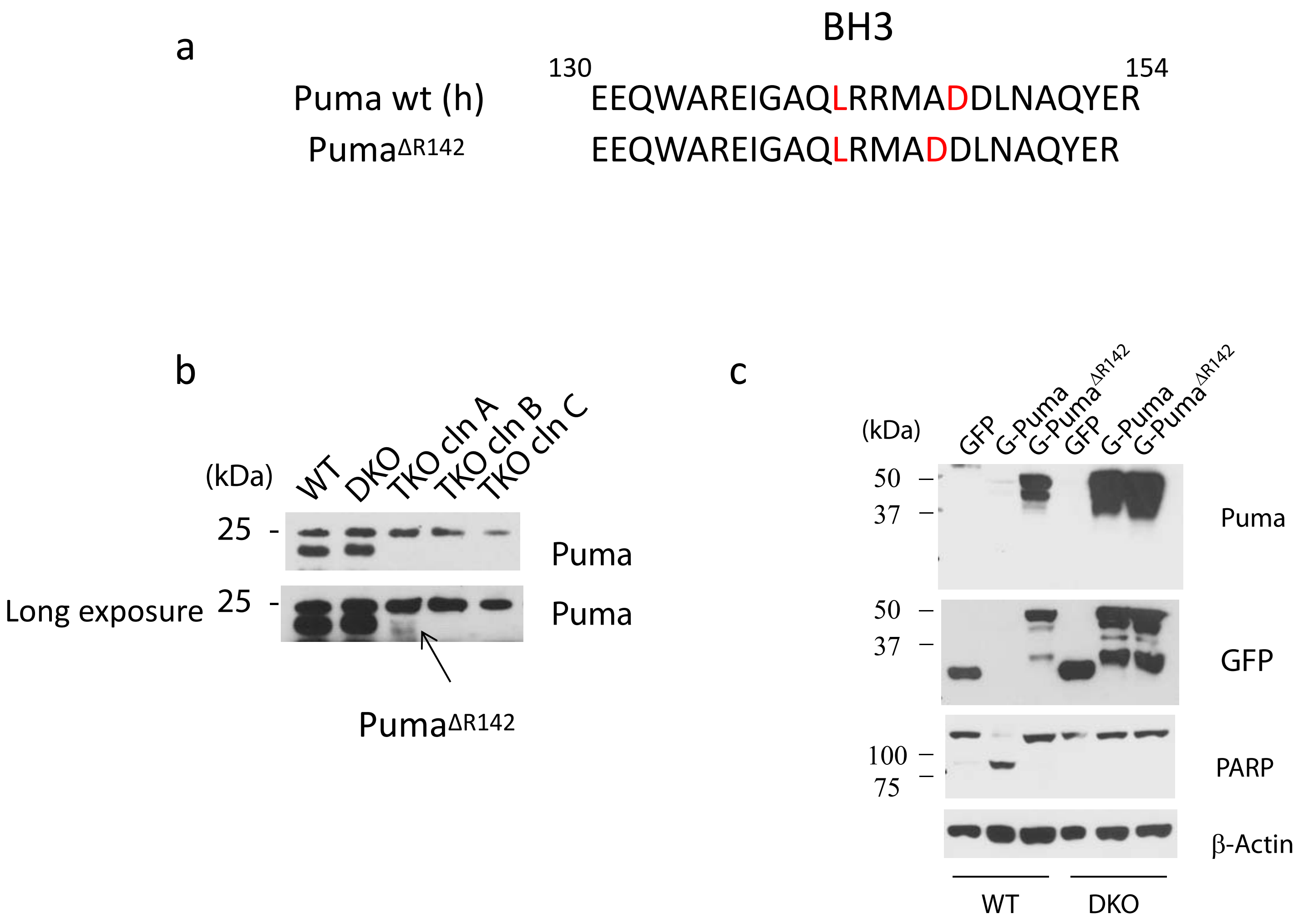

Fig. S2. Inactivation of Puma by a 3 bp in-frame mutation in TKO clone A.

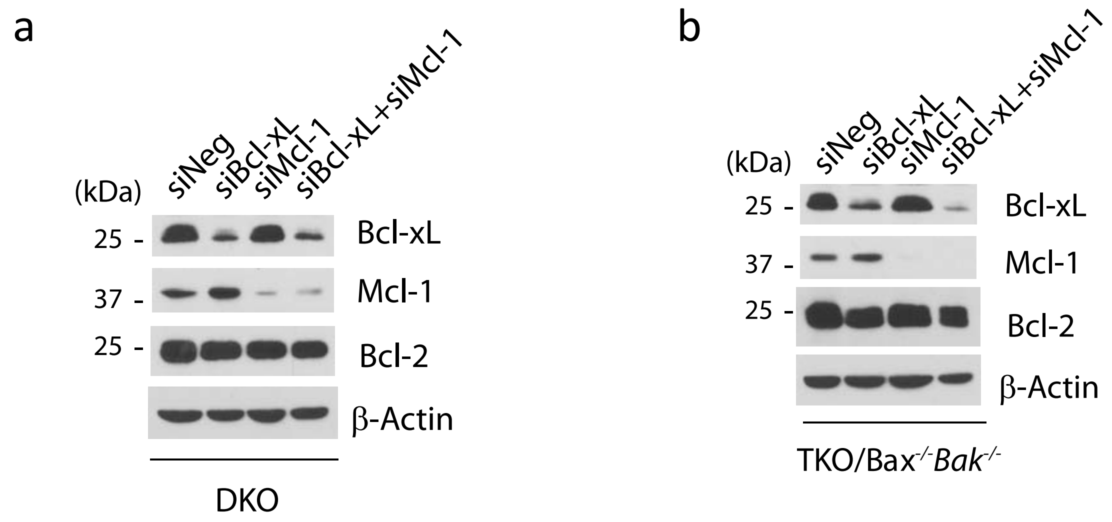

Fig. S3. SiRNA knockdown of Mcl-1 and/or Bcl-xL in DKO and TKO/*Bax*$^{-/-}$*Bak*$^{-/-}$ cells.

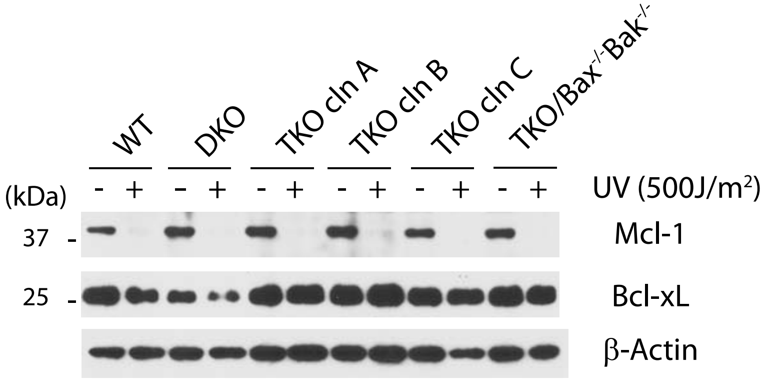

Fig. S4. Elimination of Mcl-1 in TKO cells following UV treatment.

```
WT_Mcl-1               TGGAGTTGGTCGGGGAATCTGGTAATAACACCAGTACGG---------------------
HexaKO_clnA_Mcl-1      TGGAGTTGGTCGGGGAATCTGGTAATAACACCAGTACGGGTTCAGGTCGCCCTCGATCAG

WT_Mcl-1               -------------------------------------------------ACGGGTCACTA
HexaKO_clnA_Mcl-1      GAAGTGGCCCGC..................GACGCAAATGGGCGGTAGGCGTGTACGGTGGACGGGTCACTA

WT_Mcl-1               CCCTCGACGCCG
HexaKO_clnA_Mcl-1      CCCTCGACGCCG
```

Fig. S5. Mutations of the targeted Mcl-1 locus in HexaKO clone A

```
WT_p53           GACCTGGGTCTTCAGTGAACCATTGTTCAATATCGTCCGGG-------------------
QKO_clnA_p53A    GACCTGGGTCTTCAGTGAACCATTGTTCAATATCGTCCGGGCATGTGCCAAGGCCCTGGG
QKO_clnA_p53B    GACCTGGGTCTTCAGTGAACCATTGTTCA-------------------------------
QKO_clnB_p53     GACCTGGGTCTTCAGTGAACCATTGTTCAATATC--------------------------

WT_p53           ------------------------------------------------------------
QKO_clnA_p53A    GCAGGAACAACTGAGAGGAGTCTGCTAAGGCTGGAGTGCAGAATCCAGGAGAGCGGTGTC
QKO_clnA_p53B    ------------------------------------------------------------
QKO_clnB_p53     ------------------------------------------------------------

WT_p53           ---------------GACAGCATCAAATCATCCATTGCTTGGGACGGCAAGGGGGACTGT
QKO_clnA_p53A    TCAATATCGTCCGGGGACAGCATCAAATCATCCATTGCTTGGGACGGCAAGGGGGACTGT
QKO_clnA_p53B    -----------------------CAAATCATCCATTGCTTGGGACGGCAAGGGGGACTGT
QKO_clnB_p53     ---------------------ATCAAATCATCCATTGCTTGGGACGGCAAGGGGGACTGT

WT_p53           AGATG
QKO_clnA_p53A    AGATG
QKO_clnA_p53B    AGATG
QKO_clnB_p53     AGATG
```

Fig. S6. Mutations of the targeted p53 locus in QKO clones

Supplementary Table S1. Cell lines generated in this study.

Cell name	Gene	Clone #	Parental clone	Exon targeted	Edited alleles	Mutations
(Bax/Bak) DKO	Bax	11	HCT116 wt	4	1,2	2bp insertion
	Bak (Bak1)			5	1,2	1bp insertion
	Bid		HCT116 wt	3	1	46 bp deletion
				3	2	25bp deletion + 39 bp insertion (introducing a stop codon) and 34 bp insertion
TKO cln A	Bim (Bcl2L11)	7		3	1	4bp deletion
				3	2	2bp deletion
	Puma			3	1	24bp deletion removing BH3
				3	2	3bp deletion removing an Arg from BH3
	Bid		HCT116 wt	3	1	1bp insertion
				3	2	11bp deletion
TKO cln B	Bim	10		3	1,2	4bp deletion
	Puma			3	1	2bp insertion
				3	2	14bp deletion
	Bid		HCT116 wt	3	1,2	1bp insertion
TKO cln C	Bim	21		3	1,2	4bp deletion
	Puma			3	1,2	1bp deletion
PentaKO	Bax	4	TKO cln A	4	1,2	90bp deletion
	Bak (Bak1)			5	1,2	1bp insertion
HexaKO	Mcl-1	5	PentaKO	1	1,2	319bp insertion
QKO cln A	p53 (TP53)	14	TKO cln A	4	1	20bp deletion
				4	2	94bp insertion
QKO cln B	p53 (TP53)	17	TKO cln A	4	1,2	13bp deletion

Supplementary Table S2. Sequences of sgRNA and the vectors.

Gene (human)	Genome editing method	Vector	Exon targeted	sgRNA Sequence
Mcl-1	CRISPR	px330	1	GTAATAACACCAGTACGGACGGG
Bax	CRISPR	px330	4	CTGCAGGATGATTGCCGCCGTGG
Bak	CRISPR	px330	5	ACGGCAGCTCGCCATCATCGGGG
Bid	CRISPR	px330	3	GCTCATCGTAGCCCTCCCACTGG
Bid	Nickase	px335	4	CGAGGTGCCTGGCAATATTCCGG
Bid	Nickase	px335	4	CATGGACCGTAGCATCCCTCCGG
Bim	CRISPR	px330	3	GCCCAAGAGTTGCGGCGTATTGG
Puma	CRISPR	px330	3	CGCTGGGCACGGGCGACTCCAGG
Puma	Nickase	px335	3	CGTCCGCCATCCGCCGCAGCTGG
Puma	Nickase	px335	3	ACCTCAACGCACAGTACGAGCGG
p53	Nickase	px335	4	GCATCAAATCATCCATTGCT (L)
p53	Nickase	px335	4	CCCCGGACGATATTGAACAA (R)

Supplementary Table S3. Primers used for genomic sequencing.

Bak CRISPR	Bak-F	CCATGGACAGCTCAGGCAGAAC
	Bak-R	CTGGGGTACCTGGTGGCAATCTT
Bax CRISPR	Bax-F	ACCCTCCTTCAGGGAGTCAT
	Bax-R	CAGTTGAAGTTGCCGTCAGA
Bim TALEN	Bim-TL-F	CGCAAGCTGTTGACATTGTT
	Bim-TL- R	GTCTGTAGGGAGGTAGGGGC
Bim CRISPR and DN	Bim-CR-F2	GGAGCTCCCAGAAATGTGAA
	Bim-CR-R2	GAGGGTGTGAGCAGAAAAGC
	Bim-out-F	ATTATGTTTCAAGCATATTTCTTT
	Bim-out-R	CCTTTAACTTTGACCTAAAATGCA
Puma CR and DN	PumaDN-F	TCGTACTGTGCGTTGAGGTC
	Puma-CR-F2	TACTGTGCGTTGAGGTCGTC
	Puma-CR-R2	ACCTTCCACACTGACAAGGG
	PumaDN-F2	caCtgttcCtCCtCcccgc
	PumaDN-R2	GTCCTCAGCCCTCGCTCT
Bid CRISPR and DN	Bid-F	CTCGATGTCAACCAGCTGAAC
	Bid-R	CCCTGCCTGAACACAGATTC
	Bid –Cr-F2	GGTCCAGACAAGGGACTCAA
	Bid –Cr-R2	TGTTCTGACAACAGCTTCCG
	Bid-DN-F1	GTGTGGGAGAGGGTATCAGG
	Bid-DN-R1	CCTCGATGTCAACCAGCTG
Mcl-1 CR	Mcl-1-nest-F2	CGACTTTTGGCTACGGAGAA
	Mcl-1-nest-R2	AACTCGTCCTCCTCCTCCTC
	Mcl-1-out-F1	CCACTTCTCACTTCCGCTTC
	Mcl-1-out-R1	AATGAACCCCCTTACCTTGG
p53 Nickase	p53DN1 out F	AGGGTGTGATGGGATGGATA
	p53DN1 out R	CCCCTCTGAGTCAGGAAACA
	p53DN1 nest F	GAAGTCTCATGGAAGCCAGC
	p53DN1 nest R	TTCACCCATCTACAGTCCCC
